# Cancer of unknown primary: the hunt for its elusive tissue-of-origin – is it time to call off the search?

**DOI:** 10.1038/s41416-025-03073-7

**Published:** 2025-07-04

**Authors:** Laura Spurgeon, Claire Mitchell, Natalie Cook, Alicia-Marie Conway

**Affiliations:** 1 Department of Medical Oncology, The Christie NHS Foundation Trust, Manchester, UK; 2 Division of Cancer Sciences, School of Medical Sciences, Faculty of Biology, Medicine and Health, The University of Manchester, Manchester, UK; 3 Cancer Research UK National Biomarker Centre, The University of Manchester, Manchester, UK

**Keywords:** Cancer of unknown primary, Tumour biomarkers

## Abstract

Cancer of Unknown Primary (CUP) is an heterogenous group of metastatic cancers, for which the primary site cannot be identified despite thorough diagnostic work-up. Whilst it is a relatively rare entity, its aggressive nature, together with the paucity of effective treatments, mean it has a disproportionately high mortality rate. Advances in genomic profiling have driven research in the area, but despite this, therapeutic options and prognosis remain poor. For many years, the primary focus in CUP has been improving identification of the tissue-of-origin (TOO), with the hope that site-specific therapy will improve survival outcomes. However, the lack of conclusive evidence to support this, as well as an emerging paradigm shift in how cancers should be classified, has reignited the debate surrounding the importance of TOO. Here, we provide a comprehensive review of the ongoing relevance of TOO, within both the CUP and wider oncology landscape.

## Background

Over 8500 cases of Cancer of Unknown Primary (CUP) are diagnosed each year in the UK, whilst it accounts for 2–5% of all new cancer diagnoses worldwide [1, 2]. Although its incidence is declining, likely due to advances in diagnostic techniques identifying primary sites, it remains the sixth leading cause of cancer-related death in the UK [3], and at least fourth globally [4]. Patients presenting with disseminated disease undergo extensive investigations to determine the tissue-of-origin (TOO) if a primary site is not readily identifiable. This initial presentation, termed malignancy of unknown origin (MUO), represents 10–15% of all new cancer diagnoses in the UK [5]. For most patients with MUO, a primary site is identified or strongly suspected after appropriate investigations; for the remaining third, a diagnosis of confirmed CUP is made [6].

Determining TOO requires adequate pathological examination of the tumour tissue; guidelines recommend a stepwise approach, including morphological assessment and appropriate immunohistochemistry (IHC) for undifferentiated neoplasms and cells with unclear lineage [7, 8]. Once carcinoma has been confirmed and other treatable malignancies excluded (such as lymphoma and sarcoma), further IHC can be applied to delineate TOO [8]. Whilst IHC using tissue obtained from both primary *and* metastatic sites can be highly accurate at determining TOO, when obtained from metastatic lesions alone, the accuracy falls significantly [9]. Given biopsies in CUP are nearly always from metastatic sites and are typically poorly-differentiated, identifying TOO using standard-of-care (SoC) IHC remains challenging.

ESMO guidelines recommend clinical work-up to help determine TOO, incorporating mandatory assessments and optional investigations, which may be indicated depending on the pattern of disease, sex, and suspected primary (Table 1) [10]. If a primary site is not identified during diagnostic work-up, patients are treated as confirmed CUP, subsequently classified into favourable and poor-risk (previously unfavourable) subtypes to guide management and prognostication. Those with favourable disease have an Eastern Cooperative Oncology Group performance status 0–1, lactate dehydrogenase within normal limits, and either low volume, predominantly nodal disease, or radiological and/or histological features aligning with a primary site. These patients account for ~20% of CUP and should be treated with site-specific therapy based on the likely primary, with outcomes similar to those of the analogous tumour [11]. Although increasingly more patients can be classified as favourable, the majority fall within the poor-risk subtype, having a high-burden of disease with visceral involvement and limited treatment response.

**Table 1:** Diagnostic work-up for patients with suspected CUP (adapted from ESMO Guidelines, 2023).

Assessment	Patient Population
Thorough medical history and physical examination	All
Blood counts including blood differential test Biochemistry analyses (including ALP, LDH and albumin)	All All
IV contrast CT or MRI scan of neck, thorax, abdomen and pelvis	All
**Optional Tests Depending on Clinical and Pathological Results**	
**Tumour markers**	
1. CEA, CA19-9, CA72-4	1. Suspected GI primary
2. Chromogranin-A	2. Suspected neuroendocrine malignancy
3. PSA	3. Males with suspected prostate cancer
4. AFP, β-hCG	4. Males with suspected germ cell tumour
5. CA15-3, CA125	5. Females with suspected gynaecological primary
PET scan	Single-site or oligometastatic CUP Head and neck-like CUP
Mammography	All females
Breast MRI	Females with axillary lymphadenopathy
Brain MRI	Clinically suspected brain metastases Putative lung primary Single-site or oligometastatic CUP
Gastroscopy or colonoscopy	Putative GI primary
Bronchoscopy	Putative lung primary

^*^ALP alkaline phosphatase, *CEA* carcinogenic embryonic antigen, *CA19-9* cancer antigen 19-9, *CA72-4* cancer antigen 72-4, *PSA* prostate specific antigen, *AFP* alpha fetoprotein, *β-hCG* βeta-human chorionic gonadotrophin, *CA15-3* cancer antigen 15-3, *CA125* cancer antigen 125, *PET* position emission tomography.

Prognosis for poor-risk CUP remains dismal, with one-year survival around 20% [10, 12] and little improvement over the last decade [13]. Platinum-doublet chemotherapy remains SoC treatment for those fit enough, with taxane-containing regimens in particular offering a modest survival benefit [14]. Response rates to chemotherapy range from 20 to 40% [15] and the intent is nearly always palliative. Although prognostic biomarkers exist to identify patients who may benefit from treatment [16, 17], therapeutic options remain limited without detection of a primary site. In an attempt to progress TOO identification, facilitate access to treatments and improve outcomes, multiple TOO classifiers have been developed, although none are currently approved for use in clinical practice due to a lack of robust prospective evidence.

## Why the Tissue-of-Origin Debate Exists

The lack of validated TOO classifiers, together with inconclusive evidence regarding their benefit, has led many to question whether TOO is still pertinent in CUP. The traditional site-based classification of cancer has driven the need to identify TOO, whilst also dictating many aspects of cancer care, from the structure of oncology training to site-specific conferences and guidelines, and most importantly for CUP, how clinical trials are designed and therapeutics subsequently licensed [18]. As a result, access to many treatments is reliant upon the identification of a primary site, thereby disadvantaging the majority of patients with CUP and reinforcing the need to identify TOO. However, recent advances in genomic profiling and molecularly guided therapy (MGT), together with an evolving appreciation of common molecular landscapes across many tumour types, call into question the current site-based classification of cancer as an outdated approach, rooted in historic tradition no longer applicable to modern medicine. Furthermore, despite years of research dedicated to the identification of TOO in CUP, outcomes remain dismal [13], with only a handful of studies offering hope of progress.

Now is an appropriate time to reflect on the unwavering search for TOO in CUP, and consider whether the focus of research, clinical investigations and subsequent management should be redirected.

## Argument Against Tissue-of-Origin

### Inconclusive evidence from clinical trials

Currently there is no practice-changing evidence to support the use of TOO classifiers in CUP, despite several tools being developed. In fact, a number of studies have concluded there is no benefit to site-specific therapy over empiric chemotherapy (Table 2). In the large, randomised phase III trial of 243 patients, GEFCAPI 04, molecularly-guided predictions of TOO to tailor systemic treatment failed to improve outcomes for patients with CUP, with no significant difference in median progression free survival (mPFS) nor median overall survival (mOS) between the site-specific and SoC arms (mPFS 5.8 vs. 6.4 months respectively, HR = 0.95; mOS 10.0 vs. 10.7 months respectively, HR = 0.92) [19]. Hayashi et al. compared a SoC platinum-doublet regimen with site-specific therapy using comprehensive microarray gene expression profiling (GEP) to predict the likely primary in a phase II study [20]. They also reported no clear benefit from TOO predictions, with a one-year OS rate of 44% in this group, compared with 54.9% for those receiving empiric chemotherapy. Median OS for site-specific therapy was 9.8 months (HR = 1.03), compared with 12.5 months for the SoC arm, although mPFS was slightly longer at 5.1 vs. 4.8 months (HR = 0.88). However, they did report that those with predicted tumour types expected to respond to chemotherapy did better overall (mOS 16.7 vs. 10.6 months; HR = 0.687) [20], suggesting TOO predictions may have some prognostic benefit in a select subgroup. This finding is supported by Yoon et al. who found patients predicted to have a platinum-responsive primary site after molecular profiling using a 2000-gene expression microarray had significantly longer mPFS and mOS than those tumours where platinum-doublet chemotherapy would not be SoC (mPFS 6.4 vs 3.5 months respectively, HR = 0.47; mOS 17.8 vs. 8.3 months respectively, HR = 0.37) [21].

**Table 2:** Summary of studies investigating the benefit of site-specific therapy in CUP following TOO predictions.

Study	Phase	Number	TOO Classifier	Response Rate (Site Specific vs. SoC)
Hainsworth et al. [22]		42	92 gene RT-PCR^a^ assay	mPFS 8.5 vs 6.0 m; *p* = 0.11
Moran et al. [59]		216	DNA methylation profiling	mOS 13.6 vs. 6.0 m, HR = 3.24, 95% CI 1.42–7.38
Yoon et al. [21]	II	46	Gene expression profiling	mPFS 6.4 vs. 3.5 m; mOS 17.8 vs. 8.3 m, *p* = 0.005^b^
Hasegawa et al. [58]		122	Pathological examination	mOS 20.3 vs. 10.7 m, HR = 0.57, 95% CI 0.34–0.94
Hayashi et al. [20]	II	130	Microarray gene expression profiling	mPFS 5.1 vs. 4.8 m, HR = 0.88, 95% CI 0.59–1.33; mOS 9.8 vs 12.5 m, HR = 1.03, 95% CI 0.68–1.56
Fizazi et al. [19] (GEFCAPI-04)	III	243	Gene expression assay	mPFS 5.8 vs. 6.4 m, HR = 0.95, 95% CI 0.72–1.25; mOS 10.0 vs 10.7 m, HR = 0.92, 95% CI 0.69–1.23
Ding et al. [23]	Meta analysis	1114	Multiple	Overall PFS for site-specific therapy: HR = 0.93, 95% CI 0.74–1.17, *p* = 0.534; overall OS for site-specific therapy: HR = 0.46, 95% CI 0.55–1.03, *p* = 0.0069
Moon et al. [60]		158	Next generation sequencing	PFS and OS not reported; HR = 0.348, 95% CI 0.21–0.57
Liu et al. [61] (CUP-001)	III	182	92-gene expression assay	mPFS 9.6 vs 6.6 m, HR = 0.68, 95% CI 0.49–0.93

^a^RT-PCR reverse-transcriptase polymerase chain reaction.

^b^Comparison between predicted primary sites where platinum/taxane chemotherapy is SoC vs. predicted primary sites where platinum/taxane is not SoC.

Additionally, Hainsworth et al. reported that site-specific therapy based on a GEP assay had comparable survival outcomes to empiric chemotherapy for all comers, but patients predicted to have chemotherapy-responsive tumours responded better [22]. This is supported by a systematic review and meta-analysis of site-specific therapy in CUP which concluded there was no significant OS benefit unless patients had a predicted chemotherapy-responsive tumour type [23]. However, it is important to interpret results from these studies in the context of the CUP syndrome: many patients have aggressive cancers which do not respond to either empiric nor site-specific treatment; until recently, a number of the common predicted sites (non-small cell lung cancer (NSCLC)/hepatobiliary (HPB)/breast/ovarian cancer), have had similar site-specific and empiric chemotherapy regimens so treatment responses are likely to have been similar regardless of TOO predictions; and the sites for which TOO would significantly change treatment, such as kidney and colorectal, are in the minority [22]. Additionally, most of these studies pre-date the introduction of immune checkpoint inhibitors (ICIs) and MGT in other poor-prognostic advanced cancers, meaning they may not accurately reflect the current disparity in survival outcomes between CUP and metastatic cancers of a known primary site.

Although the studies discussed here do not provide convincing evidence to support the use of site-specific therapy in all patients with CUP, they do suggest that identifying those with treatment-responsive disease sites could be beneficial. Due to the heterogeneity within CUP, it is likely that the proportion of patients who could benefit from site-specific therapy may be too small to see a survival advantage in large clinical trials. As evidenced by early studies of epidermal growth factor receptor (EGFR) inhibitors in lung cancer, the survival advantage from erlotinib and gefitinib was not demonstrated in clinical trials until predictive EGFR mutations were identified [24]. Perhaps then, a survival advantage from site-specific therapy in an heterogenous CUP population may only be observed when predictive biomarkers are identified in each TOO-directed subgroup.

### CUP as a unique entity

The atypical pattern of metastases in poor-risk CUP remains an unexplained hallmark of the disease, and may suggest that CUP is a unique entity, behaving in an inherently different way to a putative primary. When TOO is identified, either at post-mortem autopsy or a later stage in the clinical course, it often reveals a pattern of metastases which does not correlate with traditional spread of the known primary [25, 26]. If some CUPs behave fundamentally differently from their putative primary, there may be limited value in identifying TOO, as extrapolation of the predicted disease course, response to treatment, and survival outcomes may be inaccurate and unlikely to meaningfully inform management.

Offering support to this argument, resistance to chemotherapy in poor-risk CUP is common, with clinical or radiological response observed in fewer than 25% of patients [27]. Whilst this may simply reflect the aggressiveness of the disease and empiric nature of the regimens, the lack of conclusive evidence to support site-specific therapy may suggest that CUP is a unique syndrome, heterogenous in nature, and distinct from its putative primary, and therefore unlikely to respond in a similar way to conventional treatment. If this is the case, focusing solely on identifying TOO could be a rudimentary approach to a complex syndrome, and indeed, some may argue that efforts to improve survival outcomes could be better served elsewhere.

### Classification of cancers

The argument for changing the way tumours are classified based on evolving knowledge of the molecular landscape driving tumorigenesis has gained momentum and may offer an alternative focus to improve outcomes. In fact, CUP could be the perfect model to illustrate the need for a paradigm shift in how we approach cancer. Hoadley et al. previously recommended that the traditional site-specific approach should be supplemented by molecular classification due to the development of therapeutic targets for common oncogenic drivers, and suggested that tumours should be characterised by shared alterations rather than ‘*cell-of-origin*’ alone [28]. André et al. more recently argued that the conventional site-specific approach to categorising tumours is denying patients access to potentially effective treatments [18]. Until recently, clinical trials were typically conducted sequentially, with expansion to additional tumour sites following approval in another disease group. This protracted process meant millions of patients expressing high levels of programmed death-ligand 1 (PD-L1) were unable to access ICIs until the efficacy was specifically evaluated in their disease site [18].

Furthermore, in 2009, clinical trials into the PARP inhibitor, Olaparib, began in ovarian cancer, with FDA approval in 2014. However, it wasn’t until 2018 that it was licensed for use in *BRCA1/2* mutated breast cancer, and took even longer for FDA approval in pancreatic and prostate cancer. It is estimated that since 2014, 100,000 breast and 200,000 pancreatic and prostate cancer patients who may have benefited from Olaparib died whilst awaiting site-specific approval [18]. Site-specific sequential trial design and strict approval regulations limit access to potentially effective treatments, a particular problem in CUP where patients are deemed ineligible for many drugs without identification of a primary site. Molecular-based classification of cancers, focusing on shared mutations or biomarkers, with subsequent basket trials can expedite new drug approvals and increase the number of eligible patients. It could therefore be argued that changing the way we classify tumours and license drugs may negate the need to determine TOO in CUP, focusing instead on tumour-agnostic identification of actionable alterations.

### Tumour agnostic approvals

Several treatments have received tumour-agnostic approval, reflecting the growing importance of precision oncology. In May 2017, Pembrolizumab was the first drug to receive accelerated FDA approval for mismatch repair-deficient (MMRd) or microsatellite instability-high (MSI-H) tumours, with full approval in March 2023 following three phase II clinical trials [29–31]. Although no ICIs are currently available in England for patients with CUP, Pembrolizumab is licensed for this indication elsewhere in the second-line setting [10]. Similarly, outside of England, patients with relapsed or refractory CUP with high PD-L1 expression and no alternative treatment options are eligible for ICIs [10], whilst favourable CUPs with tumours analogous to a primary site with known benefit from immunotherapy, such as NSCLC, urothelial, and head and neck cancers, can also be considered for ICIs [10, 32].

Since 2017, a number of MGTs have been licensed on a tumour-agnostic basis under FDA regulations; most recently, trastuzumab deruxtecan received accelerated approval for HER2 IHC3+ cancers based on three phase II multicentre clinical trials [33–35]. In the UK, entrectinib and larotrectinib are the only MGTs to have been approved for use in CUP [36], reflecting the disparity in access to tumour-agnostic treatments globally. Additionally, tumour-agnostic molecular alterations, such as *BRAF* V600E and *RET*, can be targeted in relapsed or refractory CUP under FDA approval [10], although, again, they are not currently available in the UK. CUP is known to have a diverse and heterogenous molecular landscape, with up to one-third of patients harbouring a potentially actionable alteration, as reported by the prospective phase II trial, CUPISCO [37]. This study also demonstrated PFS benefit of MGT over empiric chemotherapy in poor-risk, treatment-naïve CUP, and was the first randomised trial in CUP to report positive results [37], highlighting the importance of precision medicine, even within a tumour-agnostic context.

Given the number of patients with CUP who could potentially benefit from MGT, together with the expansion of drugs receiving tumour-agnostic approval, perhaps the need to identify TOO is diminishing as precision medicine alone may be sufficient to guide management. However, it is important to acknowledge that only 27% of patients randomised to the MGT arm in CUPISCO actually received targeted therapy; the remaining 73% were treated with combination chemo-immunotherapy in the absence of an actionable alteration [37]. Despite the increasing use of MGT, there remains a large proportion of patients with CUP for whom chemotherapy and/or immunotherapy will be SoC, with TOO needed to determine the most appropriate regimen.

### Summary – argument against tissue-of-origin

The main argument against the ongoing relevance of TOO stems from the lack of conclusive evidence to support site-specific therapy in CUP, although only a relatively small number of trials have sought to answer the question. Additionally, most of these trials pre-date the introduction of site-specific, targeted therapies meaning they may not accurately reflect the current use of personalised medicine and subsequent improved outcomes. Taken together with growing impetus to change the way cancer is classified and drugs are licensed, TOO may appear less relevant in the era of precision medicine. However, there are several important factors in favour of TOO which add complexity to the debate.

## Arguments For Tissue-of-Origin

### Accessibility of treatments

Although momentum for molecular-based classification of cancers may be growing, transitioning away from the traditional site-based system would require a major overhaul of current thinking, diagnostic and treatment pathways, licensing regulations, structure of oncology training and care provision, as well as targeted patient and public re-education. Given the site-based classification is unlikely to be superseded in the near future, patients with CUP will continue being disadvantaged as most anti-cancer treatments are licensed on a site-specific basis, and those with poor-risk disease remain unable to access potentially effective treatments unless recruited to early-phase clinical trials, which themselves are often limited by geographical location.

**Fig. 1:**
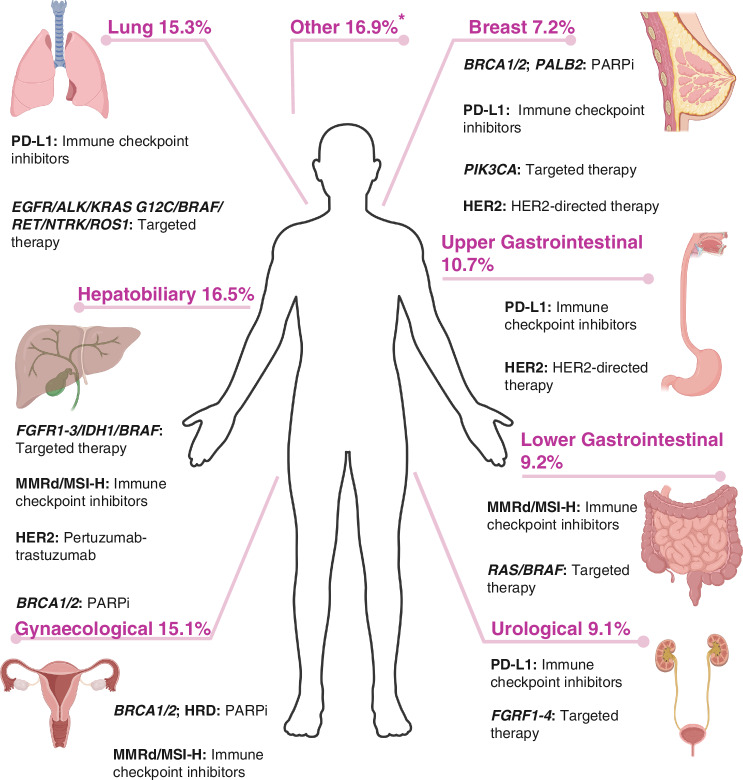
Proportion of predicted tumour types within large CUP cohorts and corresponding treatment options. Proportions represent the combined TOO classifier primary site predictions from 11 studies [19–21, 37, 48, 51–54, 86, 87] and corresponding treatment options. *Created with BioRender.com. Spurgeon, L. (2025)*. https://BioRender.com/221w770. *Other – sarcoma; lymphoma; skin/melanoma; squamous-cell carcinoma; prostate; appendix; anal **PDL1 programmed death-ligand 1, FGFR fibroblast growth factor, MMRd mismatch repair deficient, MSI-H microsatellite instability-high, HER2 human epidermal growth factor 2, BRCA breast cancer gene, PALB2 partner and localiser of BRCA2.

Effective combination therapies are becoming increasingly important in several disease sites, such as cholangiocarcinoma (chemotherapy plus durvalumab [38]), oesophageal cancer (chemotherapy plus HER2-directed therapy [39]) and lung cancer (chemotherapy plus ICIs [40]). Some of these treatments are accessible based on disease site alone, whereas others rely on predictive biomarkers to guide treatment (Fig. 1). Pathological and molecular testing, including for these predictive biomarkers, is mostly instigated based on tumour site in the UK, (although there are some exceptions, such as *NTRK* testing [41]), meaning patients without an identified primary are typically unable to access tests which serve as a gateway to potentially effective treatments. This furthers the disparity between CUP and metastatic cancers of a known site, with regard to both therapeutic options and clinical outcomes. Thus, the need to identify TOO remains necessary, given it is currently the only way to access crucial investigations and the majority of treatments, outside of experimental trials and a handful of tissue-agnostic approvals.

### Variable responses to molecularly guided therapy

Although CUPISCO did not directly evaluate the efficacy of site-specific therapy based on TOO predictions, it did suggest that treatment based on molecular alterations can improve outcomes in CUP [37], indirectly reinforcing the need to identify TOO in order to access MGT. CUPISCO also reported that response to MGT was diverse, with certain molecular targets correlating with better PFS, OS and health-related quality-of-life (HRQOL); notably, patients with *BRAF V600E* or *K106E* mutations treated with vemurafenib plus cobimetinib, and those who received pemigatinib for *FGFR1-3* mutations [37], highlighting the need for early identification and stratification of actionable alterations.

However, the significance of driver alterations and most ICI biomarkers is inextricably linked to the primary site, and therefore identifying TOO remains necessary to fully interpret the predictive implications of most cancer biomarkers. Indeed, it is the unpredictable clinical response to MGT between tumour sites sharing a common driver mutation that presents perhaps the strongest challenge to a new, molecular-based classification of cancer, and strengthens the argument for identifying TOO. A basket trial assessing the efficacy of the *BRAF* inhibitor, vemurafenib, exemplifies the importance of TOO; despite most cases carrying the *BRAF* V600E mutation, response rates varied widely; 0% in colorectal cancer, to 12% in cholangiocarcinoma, and 42% in NSCLC [42]. Additionally, TOO identification can inform potential clinically relevant resistance mechanisms; for example, melanoma patients with a *BRAF* V600E mutation are known to benefit from combination *BRAF* and *MEK* inhibitors, whilst colorectal patients with the same alteration don’t derive similar benefit [43]. Predicting response to treatment based on a single molecular alteration is challenging, and the site-specific context within which a patient receives a drug is highly relevant. Although MGT without identification of TOO has the potential to improve PFS for patients with CUP, as demonstrated by CUPISCO [37], interpreting a tumour’s molecular profile in isolation would be remiss, and instead, integrating this information within the context of TOO is necessary to appreciate the predictive and prognostic significance [28].

### Novel tissue-of-origin classifiers

In an attempt to improve identification of the primary site and subsequent survival outcomes, multiple TOO classifiers have been developed. Knowledge from large studies into multi-omics molecular profiling of known tumours, such as the landmark programme, The Cancer Genome Atlas [44], has allowed unique molecular signatures specific to the TOO to be identified and applied to CUP in several proof-of-principle studies [28]. Using artificial intelligence, classifiers are trained to detect site-specific molecular patterns using RNA, microRNA or DNA methylation profiles from known primaries, before being applied to CUP samples to predict a TOO. These approaches have 76–96% overall accuracy at predicting the origin in known tumour types, and although challenging to verify, 83–90% in CUP [45–49]. When classifier predictions are made in CUP, they are typically consistent with the clinical and histopathological features of the tumour, and in a small minority of patients for whom a dormant primary emerges later in the disease course, predictions are accurate in ~75% cases [12, 22, 50].

One challenge associated with early TOO classifiers was their reliance on tissue-based analysis, with up to 60% of molecular profiling studies in CUP failing due to lack of adequate tissue [51]. Good-quality tumour tissue is notoriously difficult to obtain in CUP; samples are often small with limited tumour content or widespread necrosis, and have typically been exhausted by multiple IHC stains [52]. To overcome this, several liquid biopsy-based techniques have been developed, predominantly using circulating cell-free DNA (cfDNA) to accurately predict TOO [53–55]. Traditionally, studies using plasma-based cfDNA analysis have focused on screening and early-detection, despite the sensitivity of cfDNA identification being higher in advanced disease [56]. The GRAIL-Galleri multi-cancer early detection test, based on cfDNA methylation analysis, was able to detect a ‘cancer signal origin’ with 97% accuracy, directing clinical work-up and resolving a primary cancer diagnosis in less than 3 months [57]. Although it wasn’t evaluated in a CUP population, it offers support for blood-based TOO classifiers, particularly as studies have shown the GRAIL test can outperform both whole genome- and targeted- sequencing at predicting TOO [54].

CUPiD, a novel machine-learning classifier tool developed for TOO predictions based on cfDNA methylation profiling, has also recently been developed specifically for CUP with promising results and a potential clinical turnaround time of three weeks [52]. CUPiD was trained on 29 tumour classes, demonstrating overall sensitivity of 84.6% and TOO accuracy of 96.8% in known tumour types [52]. When applied to a cohort of 41 patients with CUP, TOO predictions were made in 78% of cases, with 88.5% accuracy based on subsequent clinical confirmation of the primary site [52]. Interestingly, the most common predicted sites were hepatobiliary and female genital-tract, with all tumour types identified warranting either ICIs or MGT as first- or second-line treatment, highlighting the importance of detecting TOO to avoid ineffective empiric chemotherapy [52].

Although further work is needed to validate CUPiD and other blood-based TOO classifiers, they offer the potential to minimise invasive repeat biopsies, overcome challenges associated with tissue-based analysis, expedite a precise diagnosis, and facilitate access to site-specific therapy.

### Benefit of tissue-of-origin predictions

Several other studies have also sought to determine the potential benefit of TOO predictions. Hasegawa et al. retrospectively reviewed IHC panels from 122 patients with poor-risk CUP to assign a TOO [58]. For those receiving site-specific therapy based on pathology-based predictions, mOS was significantly longer (20.3 vs. 10.7 months, HR = 0.57) [58]. Additionally, Moran et al. reported that TOO predictions based on comprehensive DNA methylation profiling improved clinical outcomes for patients with CUP by facilitating access to site-specific therapy [59]. In this study, 188 patients had their tumour classified by the diagnostic assay, EPICUP, with 31 receiving site-specific treatment. Median OS in this group was 13.6 months; in contrast, those who received empiric chemotherapy had a mOS of just 6 months [59].

In a recent study using machine-learning for a next generation sequencing-based classifier (OncoNPC [60]), Moon et al. reported high-confidence TOO predictions in 41.2% of 971 CUP cases, with >2-fold increase in the number of patients able to receive genomically-guided therapy [60]. For patients receiving treatments in-line with their predicted primary site, survival outcomes were also significantly improved [60]. Furthermore, a single-site, randomised trial (CUP-001) using a 90-gene expression assay in 182 treatment-naïve patients with CUP reported a significantly longer mPFS in those receiving site-specific therapy, compared with empiric chemotherapy (9.6 vs. 6.6 months respectively; HR = 0.68) [61]. Despite progress in the area, TOO classifiers remain limited by the number of tumour types they have been ‘trained’ on, paucity of prospective clinical trials demonstrating improved outcomes from site-specific therapy, and lack of primary tissue which makes validating predictions problematic [62, 63].

With future advances in technology likely to refine existing TOO classifiers, it is conceivable that a validated tool may soon be approved for use in SoC. However, a large, prospective, multi-site randomised trial reporting positive results in favour of TOO classifier predictions and subsequent site-specific therapy is still likely to be needed to change practice.

### Emerging favourable subgroups in CUP

Evidence from emerging CUP subgroups also offers support for the importance of TOO. Rassy et al. describe three new subgroups – CUP with a colon-cancer profile (CUP-CCP), CUP with a lung-cancer profile (CUP-LCP), and CUP with a renal cell carcinoma profile (CUP-RCC) – which are increasingly being recognised as distinct, favourable CUP entities [64]. Indeed, ESMO guidelines now include diagnostic algorithms based on IHC and molecular profiling to assist in the identification of these subgroups [10], reflecting their growing importance. All subgroups have markedly different treatments to the empiric chemotherapy used in CUP, with improved outcomes when treated according to the analogous primary [64].

Outcomes for patients with CUP-CCP treated with appropriate colorectal regimens are comparable to those with metastatic colorectal cancer, and superior to patients with CUP treated with empiric chemotherapy [64]. Several studies have also demonstrated both a PFS and OS benefit for patients with CUP-CCP treated with appropriate colorectal chemotherapy [50, 65, 66]. Patients with CUP-LCP were previously classified as poor-prognosis and treated with empiric platinum-doublet chemotherapy. However, MGT and ICIs have transformed treatment options and outcomes in lung cancer, particularly NSCLC, meaning platinum-doublet is now rarely the most appropriate first-line treatment. Accurate identification of CUP-LCP with subsequent access to MGT and ICIs based on molecular profiling and PD-L1 status has the potential to improve outcomes for a large subgroup of CUP patients, given lung is the most commonly identified primary tumour at autopsy [67].

CUP-RCC is often missed on IHC as RCC-specific analysis is not performed unless there is strong clinical suspicion [68]. Whilst thought to be a relatively rare entity, its incidence has been estimated at 6–9% based on molecular analysis of two large CUP cohorts [37, 69]. Indeed, molecular profiling can be useful in identifying CUP-RCC as several alterations are strongly associated with different RCC subtypes, such as *VHL* inactivation and *MET* amplification in clear cell and papillary respectively. Given RCC is not a chemotherapy-responsive cancer, the identification of renal TOO is clearly important to facilitate access to appropriate management. Multiple studies have confirmed that CUP-RCC treated with empiric CUP chemotherapy regimens typically fail to show any response [68, 70, 71], whilst those treated with appropriate surgical resection, ICIs and/or tyrosine kinase inhibitors have outcomes far beyond those seen in CUP [64]. The disparity between empiric CUP and renal-specific treatment regimens, together with the unlikelihood of response to traditional chemotherapy, exemplifies the need to identify TOO in CUP to avoid administering ineffective and potentially harmful treatments to patients who will derive little to no benefit.

### Diagnostic challenges

CUP is inherently difficult to diagnose, and many patients won’t have a primary site identified due to diagnostic limitations, either through atypical clinical presentation or inadequate IHC markers. It remains a diagnosis-of-exclusion, with significant challenges posed to pathologists, radiologists and clinicians. However, its incidence is declining, ~30% over the last decade, with a further 13% fall expected by 2040 [1], no doubt due to improving diagnostic techniques and increased awareness of previously unrecognised subgroups, each with distinct radiological, histopathological and/or molecular patterns.

Two of the most common CUP differential diagnoses, NSCLC and intrahepatic-cholangiocarcinoma (iCCA), exemplify the diagnostic challenges in CUP. Thyroid transcription factor 1 (TTF-1) positivity is typically associated with NSCLC [72], however, several studies have shown that TTF-1 staining in the metastatic setting does not confirm lung origin [73, 74], and in fact, ~20–40% of poorly-differentiated lung adenocarcinomas are TTF-1 negative [75]. Awareness of the prevalence of TTF-1 negative NSCLC and the need for additional markers, such as *SMARCA4* [76], to confirm a lung primary could increase the likelihood of this TOO being assigned to patients who may otherwise receive a diagnosis of CUP [10]. TTF-1 negativity is a poor prognostic indicator in NSCLC [72], which requires site-specific treatment to improve survival [77]. Recognising TTF-1 negative NSCLC as a rare but emerging diagnosis may increase TOO identification in CUP and facilitate access to appropriate treatments.

Differentiating between CUP with liver metastases and iCCA in a CK7+ adenocarcinoma without a discriminating IHC profile is challenging and often requires a specific iCCA-focused radiology review, which is not always performed during SoC investigations, meaning the diagnosis is often overlooked [76]. In a single-centre UK study, 34% of patients with liver-involved provisional-CUP fulfilled the criteria for iCCA based on retrospective radiological review [78]. Molecular profiling is likely to aid the diagnosis of iCCA given its distinct aberrations; however, until this is routinely accessible to patients and without specialist radiological input, iCCA will remain an overlooked, and often missed, diagnosis in CUP. Previously a poor-prognostic cancer type, iCCA now has effective first- and second-line treatment options [79], only accessible to patients with an assigned TOO.

Diagnostic challenges in CUP perpetuate the inability to assign TOO, thereby denying patients access to site-specific pathological and molecular testing, as well as personalised treatment. Evidence from increasingly recognised favourable subgroups (CUP-CCP, CUP-LCP, CUP-RCC), as well as rarer emerging subgroups (TTF1-negative NSCLC, iCCA) suggest maximising diagnostic capability to identify TOO offers patients a potential survival advantage.

### Psychosocial benefits of tissue-of-origin

**Fig. 2:**
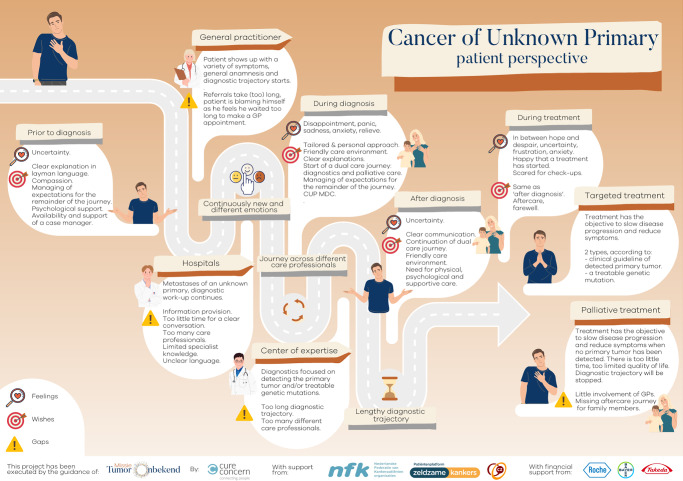
Patient perspective of the diagnostic and treatment pathway in Cancer of Unknown Primary (used with permission from World Cup Alliance [88]).

Compounding poor survival, patients with CUP are known to have significant psychological burden [80], namely anxiety, depression and somatisation disorders [81]. Patients report insufficient understanding of their diagnosis, uncertainty about prognosis, and lack of meaningful support, intensifying feelings of hopelessness which are well documented in patients with advanced cancer but particularly prominent in CUP (Fig. 2), reported in ~40% of patients [82–84]. Whilst identifying TOO may not entirely alleviate those emotions, it is likely to relieve some of the diagnostic uncertainty associated with CUP, increase patients’ understanding of their disease, prognosis and treatment options [84], and facilitate access to support services which are provided on a site-specific basis and often lacking in CUP due to the rarity of the disease. Much has been written about the ‘power of diagnosis’ and the implications it can have, from dictating clinical pathways to influencing patients’ self-perception and wellbeing [85]. Perhaps offering patients with CUP a definitive diagnosis following identification of TOO could have important HRQOL and psychosocial benefits, which may be overlooked if the importance of detecting the primary site is determined solely by outcomes from clinical trials, rather than considering a more holistic, patient-centric approach.

### Summary – Argument for tissue-of-origin

Failing to identify TOO is disadvantaging the majority of patients with CUP through drug inaccessibility, reliance on ineffective empiric chemotherapy, and increasing psychological burden. Outcomes from emerging favourable subgroups treated with site-specific therapy, as well as recent classifier developments, are encouraging and offer strong support in favour of continuing to identify TOO.

## Conclusion

Clinical trials have shown variable benefit of site-specific therapy in CUP, although recent studies have reported positive results, possibly due to refinement of TOO classifiers and treatment advances in metastatic cancers of known primary sites. However, support for the relevance of TOO goes beyond trial data; without identification of a primary site, the majority of patients will continue being disadvantaged due to drug licensing regulations, whilst diagnostic uncertainty compounds their psychological burden. Even with the emerging impetus for a new molecular-based classification of cancer and advances in precision medicine, interpreting the predictive and prognostic significance of genomics in isolation of TOO appears insufficient. Whilst CUP may indeed have unique biological characteristics, for now, determining TOO remains important to facilitate access to appropriate treatments, improve survival outcomes, enhance patients’ psychosocial wellbeing, and reduce the disparity between CUP and other metastatic cancers. We would therefore conclude that the search for the elusive TOO must go on.

## References

[1] Cancer Research UK [CRUK]. Cancer of unknown primary (online). Available via: https://www.cancerresearchuk.org/about-cancer/cancer-unknown-primary-cup/about (2024). Accessed January 2025.

[2] Qaseem A, Usman N, Jayaraj JS, Janapala RN & Kashif T. Cancer of Unknown Primary: A Review on Clinical Guidelines in the Development and Targeted Management of Patients with the Unknown Primary Site. Cureus (2019). 10.7759/cureus.5552. 10.7759/cureus.5552 PMC6820325 31695975

[3] Cancer Research UK [CRUK]. Cancer of unknown primary (online). Available via: https://www.cancerresearchuk.org/about-cancer/cancer-unknown-primary-cup/survival (2017). Accessed January 2025.

[4] Alshareeda AT, Al-Sowayan BS, Alkharji RR, Aldosari SM, Al Subayyil AM, Alghuwainem A. Cancer of Unknown Primary Site: Real Entity or Misdiagnosed Disease? J Cancer. 2020;11:3919–31. 32328196 10.7150/jca.42880 PMC7171483

[5] National Institute for Clinical Excellence [NICE]. Metastatic malignant disease of unknown primary origin in adults: diagnosis and management. Available via: https://www.nice.org.uk/guidance/cg104(2010). Accessed January 2025. 39808017

[6] Oien KA & Dennis JL. Diagnostic work-up of carcinoma of unknown primary: from immunohistochemistry to molecular profiling. Ann Oncol. 10.1093/annonc/mds357 (2012). 10.1093/annonc/mds357 22987975

[7] Selves J, Long-Mira E, Mathieu M-C, Rochaix P, Ille M. Immunohistochemistry for Diagnosis of Metastatic Carcinomas of Unknown Primary Site. Cancers (Basel). 2018;10:108. 29621151 10.3390/cancers10040108 PMC5923363

[8] Royal College of Pathologists. Standards and datasets for reporting cancers: Dataset for histopathological reporting of cancer of unknown primary and malignancy of unknown primary origin. Available via: https://www.rcpath.org/static/555302b1-8b11-4d8a-a24431d0693d3287/G167-Dataset-for-histopathological-reporting-of-cancer-of-unknown-primary-and-malignancy-of-unknown-primary-origin (2024). Accessed January 2025.

[9] Andersson GG, Weiss LM. Determining tissue of origin for metastatic cancers: meta-analysis and literature review of immunohistochemistry performance. Appl Immunohistochem Mol Morph. 2010;18:3–8. 10.1097/PAI.0b013e3181a75e6d 19550296

[10] Kramer A, Bochtler T, Pauli C, Baciarello G, Delorme S, Hemminki K, et al. Cancer of unknown primary: ESMO Clinical Practice Guideline for diagnosis, treatment and follow-up. Ann Oncol. 2023;34:228–46. 36563965 10.1016/j.annonc.2022.11.013

[11] Hainsworth JD, Fizazi K. Treatment for patients with unknown primary cancer and favorable prognostic factors. Semin Oncol. 2009;36:44–51. 19179187 10.1053/j.seminoncol.2008.10.006

[12] Varadhachary GR, Raber MN. Cancer of Unknown Primary Site. N. Engl J Med. 2014;371:757–65. 25140961 10.1056/NEJMra1303917

[13] Binder C, Matthes KL, Korol D, Rohrmann S, Moch H. Cancer of unknown primary – Epidemiological trends and relevance of comprehensive molecular profiling. Cancer Med. 2018;7:4814–24. 30019510 10.1002/cam4.1689 PMC6144156

[14] Lee J, Hahn S, Kim D-W, Kim J, Kang SN, Rha SY, et al. Evaluation of survival benefits by platinums and taxanes for an unfavourable subset of carcinoma of unknown primary: a systematic review and meta-analysis. Br J Cancer. 2013;108:39–48. 23175147 10.1038/bjc.2012.516 PMC3553519

[15] Amela EY, Lauridant-Philippin G, Cousin S, Ryckewaert T, Adenis A, Penel N. Management of ‘unfavourable’ carcinoma of unknown primary site: synthesis of recent literature. Crit Rev Oncol Hematol. 2012;84:213–23. 22503530 10.1016/j.critrevonc.2012.03.003

[16] Harvey S, Stares M, Scott JA, Thottiyil TJV, Conway A-M, Haigh R, et al. Biomarkers of systemic inflammation provide additional prognostic stratification in cancers of unknown primary. Cancer Med. 10.1002/cam4.6988 2024. 38404120 10.1002/cam4.6988 PMC10895198

[17] Stares M, Purshouse K, Knowles G, Haigh R, Irvine J, Gatenby A, et al. Characterisation and outcomes of patients referred to a regional cancer of unknown primary team: a 10-year analysis. Br J Cancer. 2021;6:1503–10. 10.1038/s41416-021-01544-1 PMC8608886 34489587

[18] Andre F, Rassy E, Marabelle A, Michiels S, Besse B. Forget lung, breast or prostate cancer: why tumour naming needs to change. Nature. 2024;626:26–9. 38347121 10.1038/d41586-024-00216-3

[19] Fizazi K, Maillard A, Penel N, Baciarello G, Allouache D, Daugaard G, et al. LBA15_PR – A phase III trial of empiric chemotherapy with cisplatin and gemcitabine or systemic treatment tailored by molecular gene expression analysis in patients with carcinomas of an unknown primary (CUP) site (GEFCAPI 04). Ann Oncol. 2019;30:851.

[20] Hayashi H, Takiguchi Y, Minami H, Akiyoshi K, Segawa Y, Ueda H, et al. Site-Specific and Targeted Therapy Based on Molecular Profiling by Next-Generation Sequencing for Cancer of Unknown Primary Site: A Nonrandomised Phase 2 Clinical Trial. JAMA Oncol. 2019;6:1931–8. 10.1001/jamaoncol.2020.4643 PMC7563669 33057591

[21] Yoon HH, Foster NR, Meyers JP, Steen PD, Visscher DW, Pillai R, et al. Gene expression profiling identifies responsive patients with cancer of unknown primary treated with carboplatin, paclitaxel, and everolimus: NCCTG N0871 (alliance). Ann Oncol. 2016;27:339–34. 26578722 10.1093/annonc/mdv543 PMC4907341

[22] Hainsworth JD, Schnabel CA, Erlander MG, Haines DW, Greco FA. A Retrospective Study of Treatment Outcomes in Patients With Carcinoma of Unknown Primary Site and a Colorectal Cancer Molecular Profile. Clin Colorectal Cancer. 2012;11:112–8. 22000811 10.1016/j.clcc.2011.08.001

[23] Ding Y, Jiang J, Xu J, Chen L, Zheng Y, Jiang W, et al. Site-specific therapy in cancers of unknown primary site: a systemic review and meta-analysis. ESMO Open. 2022;7:100407. 35248824 10.1016/j.esmoop.2022.100407 PMC8897579

[24] Lynch TJ, Bell DW, Sordella R, Gurubhagavatula S, Okimoto RA, Brannigan BW, et al. Activating Mutations in the Epidermal Growth Factor Receptor Underlying Responsiveness of Non-Small Cell Lung Cancer to Gefitinib. N. Engl J Med. 2004;350:2129–39. 15118073 10.1056/NEJMoa040938

[25] Le Chevalier T, Cvitkovic E, Caille P, Harvey J, Contesso G, Speilmann M, et al. Early metastatic cancer of unknown primary origin at presentation. A clinical study of 302 consecutive autopsied patients. Arch Intern Med. 1988;148:2035–9. 3046543

[26] Nystrom JS, Weiner JM, Heffelfinger-Juttner J, Irwin LE, Bateman JR, Wolf RM. Metastatic and histological presentations in unknown primary cancer. Semin Oncol. 1977;4:53–58. 841350

[27] Pentheroudakis G, Briasoulis E, Karavassalis V, Fountzilas G, Xeros N, Samelis G, et al. Chemotherapy for patients with two favourable subsets of unknown primary carcinoma: Active, but how effective? Acta Oncol. 2005;44:155–60. 15788295 10.1080/02841860510029554

[28] Hoadley KA, Yau C, Hinoue T, Wolf DM, Lazar AJ, Drill E, et al. Cell-of-Origin Patterns Dominate the Molecular Classification of 10,000 Tumors from 33 Types of Cancer. Cell. 2018;173:291–304. 29625048 10.1016/j.cell.2018.03.022 PMC5957518

[29] Marabelle A, Le DT, Ascierto PA, Di Giacomo AM, Jesus-Acosta AD, Delord J-P, et al. Efficacy of Pembrolizumab in Patients with Noncolorectal High Microsatellite Instability/Mismatch Repair-Deficient Cancer: Results From the Phase II KEYNOTE-158 Study. J Clin Oncol. 2020;38:1–10. 31682550 10.1200/JCO.19.02105 PMC8184060

[30] Le DT, Diaz LA Jr, Kim TW, Cutsem EV, Geva R, Jäger D, et al. Pembrolizumab for previously treated, microsatellite instability-high/mismatch repair-deficient advanced colorectal cancer: final analysis of KEYNOTE-164. Eur J Cancer. 2023;186:185–95. 37141828 10.1016/j.ejca.2023.02.016

[31] Geoerger B, Kang HJ, Yalon-Oren H, Yalon-Oren M, Marshall LV, Vezina C, et al. Pembrolizumab in paediatric patients with advanced melanoma or a PD-L1 positive, advanced, relapsed, or refractory solid tumour or lymphoma (KEYNOTE-051): interim analysis of an open-label, single-arm, phase 1-2 trial. Lancet Oncol. 2020;21:121–33. 31812554 10.1016/S1470-2045(19)30671-0

[32] Marcus L, Lemery SJ, Keegan P, Pazdur R. FDA Approval Summary: Pembrolizumab for the Treatment of Microsatellite Instability-High Solid Tumours. Clin Cancer Res. 2019;25:3753–8. 30787022 10.1158/1078-0432.CCR-18-4070

[33] Meric-Bernstam F, Makker V, Oaknin A, Oh D-Y, Banerjee S, González-Martín A, et al. Efficacy and Safety of Trastuzumab Deruxtecan in Patients With HER2-Expressing Solid Tumours: Primary Results From the DESTINY-PanTumor02 Phase II Trial. J Clin Oncol. 2024;42:47–58. 37870536 10.1200/JCO.23.02005 PMC10730032

[34] Smit EF, Felip E, Uprety D, Nagasaka M, Nakagawa K, Rodríguez LP-A, et al. Trastuzumab deruxtecan in patients with metastatic non-small cell lung cancer (DESTINY-Lung01): primary results of the HER2-overexpressing cohorts from a single-arm, phase 2 trial. Lancet Oncol. 2024;25:439–54. 38547891 10.1016/S1470-2045(24)00064-0

[35] Raghav K, Siena S, Takashima A, Kato T, den Eynde MV, Pietrantonio F, et al. Trastuzumab deruxtecan in patients with HER2-positive advanced colorectal cancer (DESTINY-CRC02): primary results from a multicentre, randomised, phase 2 trial. Lancet Oncol. 2024;25:1147–62. 39116902 10.1016/S1470-2045(24)00380-2

[36] Genomics Education Programme. Patient with carcinoma of unknown primary and somatic (tumour) NTRK rearrangement. Available via: https://www.genomicseducation.hee.nhs.uk/genotes/in-the-clinic/results-patient-with-carcinoma-of-unknown-primary-and-somatic-tumour-ntrk-rearrangement/ (2023). Accessed February 2025.

[37] Kramer A, Bochtler T, Pauli C, Shiu K-K, Cook N, Janoski de Menzes J, et al. Molecularly guided therapy versus chemotherapy after disease control in unfavourable cancer of unknown primary (CUPISCO): an open-label, randomised, phase 2 study. Lancet. 2024;404:527–39. 39096924 10.1016/S0140-6736(24)00814-6

[38] Vogel A, Bridgewater J, Edeline J, Kelley RK, Klümpen HJ, Malka D, et al. Biliary tract cancer: ESMO Clinical Practice Guidelines for diagnosis, treatment and follow up. Ann Onc. 2023;34:127–124. 10.1016/j.annonc.2022.10.506 36372281

[39] Obermannová R, Alsina M, Cervantes A, Leong T, Lordick F, Nilsson M, et al. Oesophageal cancer: ESMO Clinical Practice Guidelines for diagnosis, treatment and follow up. Ann Onc. 2022;33:992–1004. 10.1016/j.annonc.2022.07.003 35914638

[40] Hendricks LE, Kerr KM, Menis J, Mok TS, Nestle U, Passaro A, et al. Non-oncogene-addicted metastatic non-small-cell lung cancer: ESMO Clinical Practice Guidelines for diagnosis, treatment and follow up. Ann Onc. 2023;34:358–76. 10.1016/j.annonc.2022.12.013 36669645

[41] Genomics Education Programme. Genomic testing supports next-generation cancer drugs. Available via: https://www.genomicseducation.hee.nhs.uk/blog/genomic-test-supports-next-generation-cancer-drugs/ (2021). Accessed February 2025.

[42] Hyman DM, Puzanov I, Subbiah V, Faris JE, Chau I, Blay J-Y, et al. Vemurafenib in Multiple Nonmelanoma Cancers with BRAF V600 Mutations. N. Engl J Med. 2015;378:726–36. 10.1056/NEJMoa1502309 PMC4971773 26287849

[43] Barras D. BRAF Mutation in Colorectal Cancer: An Update. Biomark Cancer. 2015;7:9–12. 26396549 10.4137/BIC.S25248 PMC4562608

[44] National Human Genome Research Institute. The Cancer Genome Atlas. Available via: https://www.genome.gov/Funded-Programs-Projects/Cancer-Genome-Atlas (2020). Accessed January 2025.

[45] Pillai R, Deeter R, Rigl CT, Nystrom JS, Miller MH, Buturovic L, et al. Validation and Reproducibility of a Microarray-Based Gene Expression Test for Tumour Identification in Formalin-Fixed, Paraffin-Embedded Specimens. J Mol Diagn. 2011;13:48–56. 21227394 10.1016/j.jmoldx.2010.11.001 PMC3070545

[46] Kerr SE, Schnabel CA, Sullivan PS, Zhang Y, Singh V, Carey B, et al. Multisite validation study to determine performance characteristics of a 92-gene molecular cancer classifier. Clin Cancer Res. 2012;18:3952–60. 22648269 10.1158/1078-0432.CCR-12-0920

[47] Meiri E, Mueller WC, Rosenwald S, Zepeniuk M, Klinke E, Edmonston TB, et al. A Second-Generation MicroRNA-Based Assay for Diagnosing Tumor Tissue Origin. Oncologist. 2012;17:801–12. 22618571 10.1634/theoncologist.2011-0466 PMC3380879

[48] Conway A-M, Mitchell C, Kilgour E, Brady G, Dive C, Cook N. Molecular characterisation and liquid biomarkers in Carcinoma of Unknown Primary (CUP): taking the ‘U’ out of ‘CUP. Br J Cancer. 2018;120:141–53. 30580378 10.1038/s41416-018-0332-2 PMC6342985

[49] Bochtler T & Kramer A. Does Cancer of Unknown Primary (CUP) Truly Exist as a Distinct Cancer Entity? Front Oncol. 9, 10.3389/fonc.2019.00402 (2019). 10.3389/fonc.2019.00402 PMC6534107 31165045

[50] Hainsworth JD, Rubin MS, Spigel DR, Boccia RV, Raby S & Quinn R, et al. Molecular Gene Expression Profiling to Predict the Tissue of Origin and Direct Site-Specific Therapy in Patients with Carcinoma of Unknown Primary Site: A Prospective Trial of the Saran Cannon Research Institute. J Clin Oncol. 31, 10.1200/JCO.2012.43.3755 (2013). 10.1200/JCO.2012.43.3755 23032625

[51] Huey RW, Shah AT, Reddi HV, Dasari P, Topham JT, Hwang H, et al. Feasibility and value of genomic profiling in cancer of unknown primary: real-world evidence from prospective profiling study. J Natl Cancer Inst. 2023;115:994–7. 37202363 10.1093/jnci/djad095 PMC10407690

[52] Conway A-M, Pearce SP, Clipson A, Hill SM, Chemi F & Slane-Tan D et al. A cfDNA methylation-based tissue-of-origin classifier for cancers of unknown primary. Nat. Commun. 17, 10.1038/s41467-024-47195-7 (2024). 10.1038/s41467-024-47195-7 PMC11024142 38632274

[53] Moss J, Magenheim J, Neiman D, Zemmour H, Loyfer N, Korach A, et al. Comprehensive human cell-type methylation atlas reveals origins of circulating cell-free DNA in health and disease. Nat Commun. 2018;9:5068. 30498206 10.1038/s41467-018-07466-6 PMC6265251

[54] Liu X, Li L, Peng L, Wang B, Lang J & Lu Q, et al. Predicting Cancer Tissue-of-Origin by a Machine Learning Method Using DNA Somatic Mutation Data. Front Genet. 11, 10.3389/fgene.2020.00674 (2020). 10.3389/fgene.2020.00674 PMC7372518 32760423

[55] Klein CA, Richards D, Cohn A, Tummala M, Lapham R, Cosgrove D, et al. Clinical validation of a targeted methylation-based multi-cancer early detection test using an independent validation set. Ann Oncol. 2021;32:1167–77. 34176681 10.1016/j.annonc.2021.05.806

[56] Bettegowda, C, Sausen, M, Leary, RJ, Kinde, I, Wang, Y, Agrawal, N et al. Detection of circulation tumor DNA in early- and late-stage human malignancies. Sci Trans Med. 6, 10.1126/scitransmed.3007094 (2014). 10.1126/scitranslmed.3007094 PMC4017867 24553385

[57] Schrag D, Beer TM, McDonnell CH, Naduald L, Dilaveri CA, Reid R, et al. Blood-based tests for multicancer early detection (MCED): a prospective cohort study. Lancet. 2023;402:1251–60. 37805216 10.1016/S0140-6736(23)01700-2 PMC11027492

[58] Hasegawa H, Ando M, Yatabe Y, Mitani S, Honda K, Masuishi T, et al. Site-specific Chemotherapy Based on Predicted Primary Site by Pathological Profile of Carcinoma of Unknown Primary Site. Clin Oncol. 2018;30:667–73. 10.1016/j.clon.2018.06.012 30196846

[59] Moran S, Martinez-Cardus A, Sayols S, Musulen E, Balana C, Estival-Gonzalez A, et al. Epigenetic profiling to classify cancer of unknown primary: a multicentre, retrospective analysis. Lancet Oncol. 2016;17:1386–95. 27575023 10.1016/S1470-2045(16)30297-2

[60] Moon I, LoPiccolo J, Baca SC, Sholl LM, Kehl KL, Hassett MJ, et al. Machine learning for genetics-based classification and treatment response prediction in cancer of unknown primary. Nat Med. 2023;29:2057–67. 37550415 10.1038/s41591-023-02482-6 PMC11484892

[61] Liu X, Zhang X, Jiang S, Mo M, Wang Q, Wang Y, et al. Site-specific therapy guided by a 90-gene expression assay versus empirical chemotherapy in patients with cancer of unknown primary (Fudan CUP-001): a randomised controlled trial. Lancet Oncol. 2024;25:1092–102. 39068945 10.1016/S1470-2045(24)00313-9

[62] Søndergaard D, Nielsen S, Pedersen CN & Besenbacher, S Prediction of Primary Tumors in Cancers of Unknown Primary. JIB. 14, 10.1515/jib-2017-0013 (2017). 10.1515/jib-2017-0013 PMC6042823 28686574

[63] Conway A-M, Mitchell C & Cook N. Challenge of the Unknown: How Can We Improve Clinical Outcomes in Cancer of Unknown Primary? J Clin Oncol. 37, 10.1200/JCO.19.00449 (2019). 10.1200/JCO.19.00449 31211603

[64] Rassy E, Parent P, Lefort F, Boussios S, Baciarello G & Pavlidis N. New rising entities in cancer of unknown primary: Is there a real therapeutic benefit*?* Crit Rev Oncol Hematol. 147, 10.1016/j.critrevonc.2020.102882 (2020). 10.1016/j.critrevonc.2020.102882 32106012

[65] Greco FA, Spigel DR, Yardley DA, Erlander MG, Ma X-M, Hainsworth JD. Molecular Profiling in Unknown Primary Cancer: Accuracy of Tissue of Origin Prediction. Oncologist. 2010;15:500–6. 20427384 10.1634/theoncologist.2009-0328 PMC3227979

[66] Varadhachary GR, Talantov D, Raber MN, Meng C, Hess KR, Jaktoe T, et al. Molecular Profiling of Carcinoma of Unknown Primary and Correlation With Clinical Evaluation. J Clin Oncol. 2008;26:4442–8. 18802157 10.1200/JCO.2007.14.4378

[67] Pentheroudakis G, Briasoulis E, Pavlidis N. Cancer of unknown primary site: missing primary or missing biology? Oncologist. 2007;12:418–25. 17470684 10.1634/theoncologist.12-4-418

[68] Greco FA, Hainsworth JD. Renal Cell Carcinoma Presenting as Carcinoma of Unknown Primary Site: Recognition of a Treatable Patient Subset. Clin Genitourin Cancer. 2018;16:893–8. 10.1016/j.clgc.2018.03.001 29610002

[69] Jacquin N, Flippot R, Masliah-Planchon J, Grisay G, Brillet R, Dupain C, et al. Metastatic renal cell carcinoma with occult primary: a multicentre prospective cohort. npj Precis Onc. 2024;8:147. 10.1038/s41698-024-00648-0 PMC11258290 39025947

[70] Honda A, Yoshimi A, Ushiku T, Shinoda Y, Kawano H, Toya T, et al. Successful control of carcinoma of unknown primary with axitinib, a novel molecular-targeted agent: a case report. Chemotherapy. 2014;60:342–5. 26288144 10.1159/000437135

[71] Overby A, Duval L, Ladekarl M, Laursen BE, Donskov F. Carcinoma of Unknown Primary Site (CUP) With Metastatic Renal-Cell Carcinoma (mRCC) Histologic and Immunohistochemical Characteristics (CUP-mRCC): Results from Consecutive Patients Treated With Targeted Therapy and Review of Literature. Clin Genitourin Cancer. 2019;17:32–37. 30268423 10.1016/j.clgc.2018.08.005

[72] Schilsky JB, Ni A, Ahn L, Datta S, Travis WD, Kris MG, et al. Prognostic impact of TTF-1 expression in patients with stage IV lung adenocarcinoma. Lung Cancer. 2017;28:205–11. 10.1016/j.lungcan.2017.03.015 PMC6423973 28625636

[73] Wang LJ, Greaves WO, Sabo E, Noble L, Tavares R, Ng T, et al. GCDFP-15 positive and TTF-1 negative primary lung neoplasms: a tissue microarray study of 381 primary lung tumors. Appl Immunohistochem Mol Morphol. 2009;17:505–11. 19620839 10.1097/PAI.0b013e3181a8e809

[74] Aversa S, Bellan C. TTF1 Expression in Pulmonary Metastatic Rectal Adenocarcinoma. Case Rep. Gastrointest Med. 2018;5:e6405135. 10.1155/2018/6405125 PMC6304559 30631609

[75] Zhang J, Gold KA, Lin HY, Swisher SG, Xing Y, Lee JJ, et al. Relationship between tumor size and survival in non-small-cell lung cancer (NSCLC): an analysis of the surveillance, epidemiology, and end results (SEER) registry. J Thorac Oncol. 2015;10:682–90. 25590605 10.1097/JTO.0000000000000456 PMC4368494

[76] Pauli C, Bochtler T, Mileshkin L, Baciarello G, Losa F, Ross JS, et al. A Challenging Task: Identifying Patients with Cancer of Unknown Primary (CUP) According to ESMO Guidelines: The CUPISCO Trial Experience. Oncologist. 2021;26:e769–e779. 33687747 10.1002/onco.13744 PMC8100559

[77] Iso H, Hisakane K, Mikami E, Suzuki T, Matsuki S, Atsumi K, et al. Thyroid transcription factor-1 (TTF-1) expression and the efficacy of combination therapy with immune checkpoint inhibitors and cytotoxic chemotherapy in non-squamous non-small cell lung cancer. Transl Lung Cancer Res. 2023;12:1850–61. 37854151 10.21037/tlcr-23-331 PMC10579824

[78] Conway A-M, Morris GC, Smith S, Vekeria M, Manoharan P, Mitchell C, et al. Intrahepatic cholangiocarcinoma hidden within cancer of unknown primary. Br J Cancer. 2022;127:531–40. 35484217 10.1038/s41416-022-01824-4 PMC9345855

[79] Vogel A, Bridgewater J, Edeline J, Kelley RK, Klümpen HJ, Malka D, et al. Biliary tract cancer: ESMO Clinical Practice Guideline for diagnosis, treatment and follow-up. Ann Oncol. 2023;34:127–40. 36372281 10.1016/j.annonc.2022.10.506

[80] Ishida K, Ando S, Komatsu H, Kinoshita S, Mori Y, Akechi T. Psychological burden on patients with cancer of unknown primary: from onset of symptoms to initial treatment. Jpn J Clin Oncol. 2016;46:652–60. 27207884 10.1093/jjco/hyw048

[81] Hyphantis T, Papadimitriou I, Petrakis D, Fountzilas G, Repana D, Assimakopoulos K, et al. Psychiatric manifestations, personality traits and health-related quality of life in cancer of unknown primary site. Psychooncology. 2013;22:2009–15. 23359412 10.1002/pon.3244

[82] Boyland L, Davis C. Patients’ experiences of carcinoma of unknown primary site: dealing with uncertainty. Palliat Med. 2008;22:177–83. 18372382 10.1177/0269216307085341

[83] Guccione L, Fisher K, Mileshkin L, Tothill R, Bowtell D, Quinn S, et al. Uncertainty and the unmet informational needs of patients with cancer of unknown primary (CUP): a cross-sectional multi-site study. Support Care Cancer. 2022;30:8217–29. 35804177 10.1007/s00520-022-07228-7 PMC9512714

[84] Wolyniec K, O’Callaghan C, Fisher K, Sharp J, Tothill RW, Bowtell D, et al. A qualitative study of patients with Cancer of Unknown Primary: Perceptions of communication, understanding of diagnosis and genomic testing, and information needs. Psychooncology. 2023;32:589–96. 36690922 10.1002/pon.6104

[85] Jutel A. Truth and lies: Disclosure and the power of diagnosis. Soc Sci Med. 2016;165:92–98. 27497860 10.1016/j.socscimed.2016.07.037

[86] Lu MY, Chen TY, Williamson DFK, Zhao M, Shady M, Lipkova J, et al. AI-based pathology predicts origins for cancers of unknown primary. Nature. 2021;594:106–10. 33953404 10.1038/s41586-021-03512-4

[87] Tothill RW, Kowalcyyk A, Rischin D, Bousioutas A, Haviv I, van Laar RK, et al. An expression-based site of origin diagnostic method designed for clinical application to cancer of unknown origin. Cancer Res. 2005;65:4031–40. 15899792 10.1158/0008-5472.CAN-04-3617

[88] World CUP Alliance. World CUP Awareness. Available via: https://www.worldcupawareness.org/webinars/ (2022). Accessed February 2025.

